# Clinical Significance of Heme Oxygenase 1 in Tumor Progression

**DOI:** 10.3390/antiox10050789

**Published:** 2021-05-17

**Authors:** Mariapaola Nitti, Caterina Ivaldo, Nicola Traverso, Anna Lisa Furfaro

**Affiliations:** Department of Experimental Medicine, University of Genoa, Via L.B. Alberti 2, 16132 Genova, Italy; mariapaola.nitti@unige.it (M.N.); caterina.ivaldo@edu.unige.it (C.I.); nicola.traverso@unige.it (N.T.)

**Keywords:** HO-1, Nrf2, cancer progression, patients, therapy, prognosis, biomarker

## Abstract

Heme oxygenase 1 (HO-1) plays a key role in cell adaptation to stressors through the antioxidant, antiapoptotic, and anti-inflammatory properties of its metabolic products. For these reasons, in cancer cells, HO-1 can favor aggressiveness and resistance to therapies, leading to poor prognosis/outcome. Genetic polymorphisms of HO-1 promoter have been associated with an increased risk of cancer progression and a high degree of therapy failure. Moreover, evidence from cancer biopsies highlights the possible correlation between HO-1 expression, pathological features, and clinical outcome. Indeed, high levels of HO-1 in tumor specimens often correlate with reduced survival rates. Furthermore, HO-1 modulation has been proposed in order to improve the efficacy of antitumor therapies. However, contrasting evidence on the role of HO-1 in tumor biology has been reported. This review focuses on the role of HO-1 as a promising biomarker of cancer progression; understanding the correlation between HO-1 and clinical data might guide the therapeutic choice and improve the outcome of patients in terms of prognosis and life quality.

## 1. Introduction

Heme oxygenase (HO) is an evolutionarily conserved enzyme that, in the presence of molecular oxygen (O_2_) and reduced nicotinamide adenine dinucleotide phosphate (NADPH), catalyzes the degradation of heme into equimolar amounts of biliverdin, carbon monoxide (CO), and free iron (Fe^2+^), releasing NADP^+^ and H_2_O [1].

Two different isoforms of HO have been described in mammalian cells (HO-1 and HO-2) and, heme oxygenase 1 (HO-1) represents the inducible form [2]. The *HMOX-1* gene maps on the human chromosome 22q12.3 [3], on a region of approximately 13,148 bp, containing five exons and four introns [4], and codifies for a 32 kDa stress protein present at low levels in physiological conditions in most mammalian tissues [2]. HO-1 induction generally occurs in response to different endogenous and exogenous stimuli, mainly related to oxidative stress and inflammation, as well as to iron metabolism imbalance [5,6,7,8]. In tissues responsible for heme metabolism, such as spleen, liver, and bone marrow, HO-1 is highly expressed [9].

The induction of HO-1 exerts pleiotropic effects. It is well known that HO-1 is involved in the adaptive response to cellular stress and in attenuating inflammation, and, in healthy cells, HO-1 maintains redox homeostasis and prevents carcinogenesis. Importantly, in cancer cells, its expression correlates with tumor growth, aggressiveness, metastatic and angiogenetic potential. Recently, a crucial role of HO-1 in tumor immune escape has also been highlighted [10,11,12,13].

All the above-mentioned functions are ascribed mainly to the activity of HO-1 metabolic products [14,15,16]. Bilirubin (BR), derived by biliverdin reduction catalyzed by biliverdin reductase (BVRA), is a powerful antioxidant [17,18,19,20], able to scavenge reactive oxygen species (ROS) [21], therefore preventing protein and lipid peroxidation [17,22,23,24]. BR plays a key role in the regulation of inflammation and adaptive immunity, exerting immunosuppressive effects and promoting immune tolerance [25,26,27]. It is important to remark that BR is an important modulator of endothelial cell activity also in the microvasculature. Indeed, BR is able to reduce leukocyte transmigration and to prevent leucocyte rolling by decreasing the expression of P- and E-selectin, VCAM, and ICAM [28,29,30,31]. CO is well known as an antiapoptotic, anti-inflammatory, antiproliferative, and anticoagulant factor [32,33,34,35] and modulates the mitogen-activated protein kinase pathway (MAPK), soluble guanylyl cyclase (sGC) and the level of intracellular cGMP [36,37,38]. HO-1-derived CO is involved in blood vessel development [39] and VEGF synthesis [37], and enhances the proliferation of endothelial cells [38]. In addition, CO is able to attenuate inflammation [40,41], acting on both T cells [42] and antigen-presenting cells [11,12,33,43]. Finally, HO-1-derived free iron induces the synthesis of the heavy chain of the iron-chelating protein ferritin and activates the membrane transporter Fe-ATPase, which is crucial for decreasing the intracellular concentration of free Fe^2+^ and for preventing ROS production through the Fenton reaction [44,45]. Notably, HO-1 overactivation, if not balanced by the induction of ferritin and iron transporters or quenching systems, can trigger ferroptosis. Indeed, in this condition, iron accumulation leads to cell death through excessive ROS production and consequent lipid peroxidation [46,47].

Among HO-1 metabolic products, only CO has been recognized to be directly involved in tumor progression, promoting cancer cell proliferation, migration, angiogenesis, and immune escape [11]. The role of HO-1-derived bilirubin in cancer biology has been hypothesized considering its pro-surviving, pro-angiogenetic, and anti-inflammatory activity [31,48]. Instead, the generation of free iron due to HO-1 activation has been proved to favor non-canonical ferroptosis and is considered a therapeutic approach.

This review touches on the relevance of HO-1 expression in cancer progression, with a particular interest in the correlation with clinical features of tumors, taking into account data from histopathological analysis of tumor specimens.

## 2. HO-1 Gene Transcription and Protein Localization

### 2.1. HO-1 Transcriptional and Post-Transcriptional Regulation

**Figure 1:**
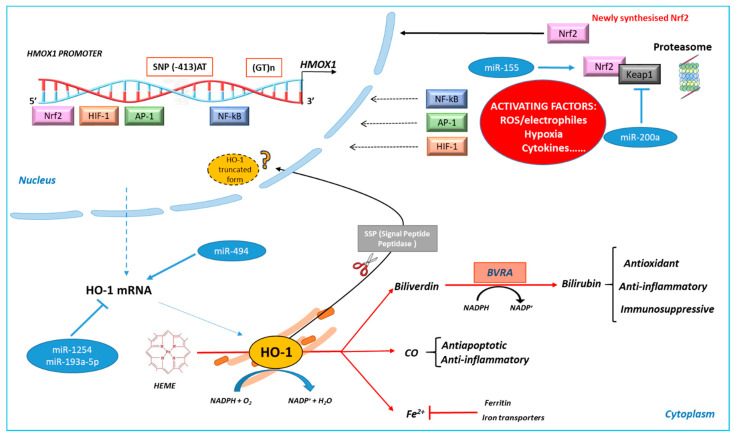
Schematic representation of heme oxygenase 1 (HO-1) activity and regulation. HO-1 induction can be regulated at the transcriptional level by several stress-related transcription factors (Nrf2, AP-1, NF-kB, and HIF-1). Two polymorphisms that modify HO-1 inducibility have been indicated. Post-transcriptional regulation can involve miRNA. HO-1 regulates intracellular heme level catalyzing its degradation into biliverdin, carbon monoxide (CO), and ferrous iron (Fe^2+^). Biliverdin is converted into bilirubin by biliverdin reductase A (BVRA). Free iron activates iron transporters and induces the expression of ferritin. HO-1 metabolic products exert pro-survival activities, as indicated. A truncated form of HO-1, formed by signal peptide peptidase (SSP) cleavage, with nuclear localization and no enzymatic activity, has been described.

The regulation of HO-1 expression occurs mainly at the transcriptional level (Figure 1). The promoter region of HO-1 contains several binding sites for different transcription factors activated in oxidative stress conditions, such as AP-1, HIF-1, NF-kB, and Nrf2 [49,50]. Thus, HO-1 is under the control of different signaling pathways. Moreover, two kinds of polymorphisms are present in its promoter region: the length of (GT)n repeats and the single nucleotide polymorphism (SNP) at the codon −413. Further, HO-1 protein levels can be regulated post-transcriptionally. Here, the main aspects of HO-1 synthesis regulation will be in brief as they are already reviewed elsewhere [51,52]; in particular, we will focus on the roles of HO-1 in cancer biology.

Among the HO-1 promoter polymorphisms, the (GT)n microsatellite repeats are crucial in modulating HO-1 expression. In particular, (GT)n polymorphisms are usually classified as short and long according to the number of the GT repeats: individuals with long (GT)n repeats show lower HO-1 inducibility due to a decreased promoter activity compared to individuals with short (GT)n repeats who have higher transcriptional activity, higher HO-1 inducibility and thus higher HO-1 levels [53]. The presence of this polymorphism correlates with the development of various pathologies, such as cardiovascular diseases, pulmonary disease [53,54,55], and cancer. However, contrasting results have been reported in different types of cancers [56].

Moreover, the SNP rs2071746 (−413A > T) polymorphism can also modulate HO-1 inducibility, being the higher HO-1 expression associated with the 413-A variant [57]. This polymorphism correlates with a reduced incidence of ischemic heart disease [58] and with graft survival after liver transplantation when present in the donor [59]. To our knowledge, only recently, the role of SNPs −413A > T in cancer risk has been analyzed by Bukowska [60]. The role of HO-1 polymorphisms in cancer will be discussed later.

The transcription factor nuclear erythroid 2-related factor-2 (Nrf2) is recognized to be the master regulator of HO-1 activation. Under nonstressed conditions, Nrf2 is bound to Kelch-like ECH-associated protein 1 (Keap1), which continuously targets Nrf2 for proteasome degradation. When cells are exposed to electrophiles and/or oxidants, Keap1 is inactivated and the newly synthetized Nrf2 is free to move into the nucleus, where it dimerizes with small Maf proteins and binds to the antioxidant/electrophile responsive elements (ARE/EpRE), leading to HO-1 gene transcription [5,61].

Of note, in cancer cells, genetic and epigenetic modifications of Nrf2/Keap1 have been described [5,62,63]. Indeed, gain-of-function mutations in Nrf2 or loss-of-function mutations in Keap1 lead to constitutive activation of Nrf2 and of its downstream target genes [5,62]. In particular, Nrf2 gain-of-function mutations have been identified in lung, head and neck, and bladder cancer, while Keap1 loss-of-function mutations have been identified in esophageal, head and neck, liver, gastric, and colorectal cancer [64]. In addition, epigenetic, especially TET-dependent demethylation of the Nrf2 promoter or Keap1 and CUL3 hypermethylation, favors Nrf2 activation, as demonstrated in lung, colorectal, and ovarian cancer [65,66,67,68].

Furthermore, HO-1 transcriptional regulation specifically involves the BTB domain and CNC homolog 1 (Bach1), a heme-binding protein that represents a major transcriptional repressor of HO-1. Indeed, Bach1 competes with Nrf2 for the binding to ARE sequences and impairs Nrf2-DNA binding activity. In response to oxidative stress, and in particular, to high levels of intracellular heme, Bach1 detaches from ARE sequences and is degraded by proteasome; in this condition, HO-1 transcription is allowed [10,69,70]. Of note, it has been demonstrated that in lung cancer metastasis, Bach1 can be stabilized in terms of protein expression and correlates with poor overall survival [71,72]. In the same works, high levels of HO-1 have been observed, meaning that the activity of HO-1 can halt Bach1 proteasomal degradation by reducing heme content. Thus, Bach1 stabilization can be observed both in the absence of Nrf2 activity or in the presence of Nrf2 activity, being dependent on the content of intracellular free heme.

Different kinase pathways (i.e., MAPKs and PI3K/AKT) are involved in HO-1 induction in cancer cells, not only by acting on Nrf2 but also by favoring Nrf2 independent HO-1 activation. p38 MAPK is responsible for Nrf2-dependent HO-1 activation in human MCF-7 breast cancer cells exposed to cadmium chloride [73] and cooperates with ERK for Nrf2-independent HO-1 activation in MKN-45 and in MKN-28 human gastric cancer cells [74]. Moreover, PI3K/AKT has been proved to be involved in HO-1 induction in SH-SY5Y neuroblastoma cancer cells in response to guanosine [75] and in cholangiocarcinoma cells treated with piperlongumine [76].

The regulation of HO-1 expression also occurs at the post-transcriptional level and microRNAs (miRNAs) play a key role. miRNAs can directly regulate HO-1 or indirectly modulate Nrf2, as already reviewed by Cheng and coworkers [77]. More recently, the involvement of miRNAs in regulating HO-1 in cancer cells has been proved. In particular, miR-155 favors lung cancer resistance to arsenic trioxide through Nrf2/HO-1 activation [78]. miR200a, in breast cancer, regulates HO-1 via Nrf2 activation by targeting Keap1 mRNA [79]. miR-1254 or miR-193a-5p, in non-small cell lung cancer (NSCLC) and prostate cancer, respectively, act on HO-1, reducing its expression and contributing to decreasing cancer cell growth [80,81]. We also demonstrated the involvement of miR494 in favoring neuroblastoma cell adaptation to oxidative stress through HO-1 up-regulation [82].

### 2.2. HO-1 Sub-Cellular and Extra-Cellular Localization

As far as HO-1 localization is concerned, HO-1 is mainly present at the endoplasmic reticulum (ER), where co-localizes with cytochrome P-450 reductase [83,84]. In addition, HO-1 can co-localize with caveolin 1/2 on plasma membrane caveolae [85] and a mitochondrial localization has been also demonstrated [86]. Of note, HO-1 can move into the nucleus, and nuclear translocation is favored by the signal peptide peptidase (SSP)-mediated intra-membrane cleavage, which leads to a C-terminal truncated form of HO-1 without catalytic activity but with transcriptional function [87,88,89]. Indeed, the truncated form of HO-1 interacts with Nrf2, increasing its stabilization [90]. Moreover, it has been demonstrated that the acetylation of the truncated form of HO-1 significantly enhances JunD-mediated AP-1 transcriptional activity leading to cancer cell proliferation, invasion, and resistance to therapy [91], indicating that post-translational modification of nuclear HO-1 plays an important role in cell proliferation, migration, and metastasis [92]. HO-1 nuclear compartmentalization is associated with cancer progression and chemoresistance, as demonstrated in chronic myeloid leukemia (CML) [93,94]; however, some opposite observations are reported in the literature [95,96,97,98]. A deeper review of the significance of HO-1 nuclear-truncated form has been recently published [92].

Furthermore, an extracellular localization of HO-1 in body fluids, including plasma, serum, milk, and cerebrospinal fluid, has been described [99,100,101]. In this context, a potential role of HO-1 as a disease biomarker has been suggested [94]. To date, the mechanisms of HO-1 release in biological fluids have not been understood. It has been hypothesized that plasma levels of HO-1 are the result of an active secretion and not the consequence of cell necrosis since it has been demonstrated, in patients with acute myocardial infarction, that HO-1 plasma levels are independent of necrosis biomarkers [102]. Interestingly, in acute kidney injury (AKI), HO-1 plasma and urinary levels parallel the level of HO-1 expression in renal tissue in response to damage [103]. Moreover, in both serum and urine, a truncated form of HO-1 was detected, suggesting that proteolytic cleavage occurs, even though the causes and consequences of this cleavage remain unknown [103]. More recently, the involvement of extracellular vesicles (EVs), such as exosomes and micro-vesicles, as potential sources of extracellular biomarkers has been considered [104,105]. In this context, HO-1 mRNA and protein have been detected in exosomes isolated from peripheral blood mononuclear cells (PMBC) of psoriasis patients [106]. Schipper and coworkers detected HO-1 protein in EVs from various human bio fluids [107]. With regard to cancer, HO-1 protein is found in EVs from the culture medium of several types of cancer cells, such as breast, lung, melanoma, and kidney [108]. However, this aspect needs further investigation.

## 3. Role of HO-1 in Cancer Progression

HO-1 overexpression has been described in several types of cancers and is associated with cancer cell proliferation, angiogenesis, invasiveness, immune escape, and resistance to therapy. However, opposite evidence has been reported as well, correlating HO-1 expression with inhibition of cancer cell proliferation, induction of apoptosis, and reduction of invasiveness; this suggests that the role of HO-1 in tumors could be tissue- and cell-specific [10].

### 3.1. HO-1 in Cancer Cell Growth, Metastasis, and Angiogenesis

The overexpression of HO-1 correlates with an increase in proliferation of cell viability in many types of cancer, such as human renal adenocarcinoma and in murine melanoma [109,110]. It favors the proliferation of malignant prostate tissues [111], pancreatic cancer, hepatoma, and lymphosarcoma [112], as well as brain and hematological cancers, as widely reviewed [11,113,114].

The acquisition of a metastatic phenotype, characterized by more aggressive features, is a key step in cancer growth and progression. In this context, HO-1 overexpression has been shown to favor metastasis development in melanoma [110], pancreatic cancer [115], oral squamous cell carcinoma [116], and prostate cancer [117]. In non-small cell lung cancer (NSCLC), the invasive and migratory abilities of cancer cells significantly increase after HO-1 overexpression, decrease after HO-1 silencing and correlate with the expression of metastasis-associated protein EGFR, CD147, and MMP9 [118]. In gastric cancer, the Nrf2-dependent HO-1 activation is involved in metastatic potential both in vitro and in vivo models [119]. Furthermore, HO-1 is involved in the epithelial-to-mesenchymal transition, a critical step in the metastasis process. Indeed, in ovarian cancer cells, HO-1 inhibition by zinc II protoporphyrin IX (ZnPPIX) down-regulates the expression of the mesenchymal markers vimentin, *N*-cadherin, and Zeb1, while up-regulates the expression of epithelial markers [120]. Consistently, it has been demonstrated that the down regulation of GRP78 increases the migration and invasiveness of colon cancer cells by the activation of Nrf2/HO-1, the induction of vimentin, and the reduction of E-cadherin expression [121].

Moreover, tumor invasiveness and metastasis development are strictly related to the stimulation of angiogenesis. In this regard, the role played by HO-1 in pathological angiogenesis of cancer is well documented both in vitro and in vivo. The up-regulation of VEGF expression in response to prostaglandin in human microvascular endothelial cells (HMEC-1) is mediated by the activation of HO-1 [122], and CO seems to be the main mediator in stimulating blood vessel formation [39]. It has been shown that HO-1 overexpression promotes angiogenesis in urothelial carcinoma cells [123] as well as in human pancreatic cancer [115]; in bladder cancer, HO-1 overexpression correlates with HIF-1α and VEGF expression [124]. Moreover, HO-1 inhibition by ZnPPIX suppresses VEGF production in GC9811-P gastric cancer cells, a cellular line characterized by high peritoneal metastatic potential [125], and in HCT-15-induced xenografts model of colorectal cancer reduces VEGF release and tumor angiogenesis [126]. In addition, inhibition of the Nrf2/HO-1 pathway by oxysophocarpine treatment suppresses the migration, the invasion potential, and the angiogenesis of oral squamous cells carcinoma [127].

### 3.2. HO-1 in Cancer Immune Escape

Recently, an important role of HO-1 in cancer immune escape has been highlighted. Indeed, HO-1 expression in infiltrating immune cells, including macrophages, dendritic cells (DC), neutrophils, natural killer cells (NK), and T and B lymphocytes, leads to their polarization toward a tumor-promoting and immunosuppressive phenotype. Moreover, HO-1 expression in cancer cells can be associated with the recruitment of specific subsets of infiltrating leucocytes and to the generation of specific cytokines that favor tumor progression.

Indeed, HO-1 expression is involved in macrophages polarization towards a pro-tolerogenic, pro-angiogenic, IL-10 producing, M2 phenotype [128], and HO-1-derived CO keeps DCs immature and modulates their cytokines secretion towards a tolerogenic phenotype [129].

In particular, it has been demonstrated that HO-1 is highly expressed in monocytes within the tumor microenvironment once they differentiate to TAMs, which indicates that HO-1 promotes their immunosuppressive function [130]. Furthermore, HO-1 detection in TAMs of prostate and breast cancers correlates with accelerated tumor growth [131,132].

Interestingly, in aggressive and metastatic prostate cancer, both in vivo and in ex vivo models, HO-1 positive macrophages were mainly detected outside the tumor tissue at the invasive zone of prostate tumors. These data suggest that extra tumor HO-1 positive macrophages could be involved in cancer aggressiveness, probably by playing a prominent role in stimulating tumor growth and metastasis [117].

Furthermore, in HO-1 overexpressing solid tumors, as well as in hematological malignancies, a high number of T regulatory cells (T_reg_) are present and act to suppress the immune response against the tumor mass [133,134,135]. For instance, in 4T1 breast cancer and in breast and melanoma bearing mice, it has been demonstrated that T_reg_ recruitment is increased in an HO-1 dependent manner [136], and HO-1 expressing T_reg_ accumulates during glioma progression [137].

Regarding the role played by HO-1 in regulating NK lymphocytes, crucially involved in the early immune response to tumor cells [138], little data are available in the literature. In a co-culture of an HO-1 positive cervical cancer cell (CCC) line and NK cells, pretreatment with various HO-1 inhibitors, tin II protoporphyrin IX (SnPPIX) and ZnPPIX, restores the expression of NKG2D, NKp30, and NKp46, markers of NK activation, and increases the production of IFN-ɣ and TNF-α, enhancing NK killing activity towards cancer cells [139]. Furthermore, we have recently demonstrated in BRAFv600 melanoma cells that HO-1 inhibition with tin mesoporphyrin IX (SnMPIX) and HO-1 siRNAdown-regulation favors cell death induced by vemurafenib, and increases NK cancer cell recognition by up-regulating B7H6 and ULBP3 ligands of NK cells [140]. To the best of our knowledge, no studies have been reported so far on the expression of HO-1 in NK cells.

### 3.3. HO-1 in the Resistance to Therapy

An important aspect of HO-1 expression in cancer cells is the gain of a resistant phenotype. It is well known that conventional anticancer treatments such as chemo- and radio- therapies can act to induce oxidative stress by increasing intracellular ROS levels [141] in order to favor apoptosis, as recently reviewed by Aggarwal and co-workers [142]. However, cancer cells, by up-regulating their antioxidant defenses, including HO-1, can counteract oxidative stress. Thus, the increase in HO-1 expression attenuates the efficacy of anticancer therapy as shown in different types of tumor where high levels of HO-1 are associated with a lower sensitivity to anticancer treatment. For instance, HO-1 overexpression is involved in resistance to chemo- and radio-therapy in central nervous system malignancies [113] and in resistance to cisplatin in hepatoma cells and ovarian cancer cells [143,144]. This aspect will be discussed later in Section 5, in the context of the possible modulation of HO-1 to favor antitumor therapies [145,146,147,148,149,150,151,152,153,154].

## 4. HO-1 Promoter Polymorphisms and Cancer Risk

As reported above, two major polymorphisms in the HO-1 promoter have been identified and linked to the modulation of HO-1 transcription: the (−413A > T) SNP and the presence of long/short (GT)n repeats. So far, no association between SNP-413 and cancers has been demonstrated, as indicated by Wang et al., who analyzed studies conducted on digestive neoplasms [155]. Moreover, recently, no prognostic significance has been shown for (−413A > T) SNP in children with acute lymphoblastic leukemia (ALL) [60].

Considering the length of GT repeats, an association has been found considering only East-Asian carriers of long (GT)n repeats, who show a high incidence of cancers in the digestive tract compared to carriers of short repeats. In fact, in Caucasian, American, and West-Asian populations, this association has not been demonstrated. Notwithstanding the small number of samples and the lack of uniformity of the studies analyzed, it seems evident that for the East-Asian populations, the presence of long (GT)n repeats is a risk factor for digestive tract cancers, probably in association with environmental factors. Indeed, in some studies, an association with alcohol consumption has been shown for the development of laryngeal squamous cell carcinoma (LSCC) for l-allele carriers in male Chinese [156]. Exposure to carcinogenic chemical compounds is a determinant to be considered; for instance, the role of smoking in male Japanese carriers of long repeats (GT)n who developed lung adenocarcinoma has been proven [157]; moreover, in asbestos-exposed Japanese subjects, the frequency of l-genotype correlates with an increased risk of developing mesothelioma [158].

An interesting study from Wu and collaborators, conducted in a cohort of patients in the area of Taiwan in which arsenic poisoning is endemic, demonstrated that (GT)n polymorphisms modify the risk of cancer due to arsenic exposure. Indeed, the risk of developing the different subtypes of arsenic-dependent tumors (skin cancer and urothelial carcinomas) is differently affected by (GT)n length. In particular, the S/S genotype carriers show a high risk of skin cancer, while no association is found for the risk of developing urothelial carcinoma among the three genotypes (S/S, L/S, and L/L) [159].

Based on this evidence, the analysis of (GT)n polymorphisms may represent a tool for evaluating an individual risk profile for a specific type of cancer, also considering the specific patient ethnicity.

## 5. HO-1 Expression, Tumor Aggressiveness, and Disease Outcome. Evidence from Immunohistochemistry

To date, the most consistent data regarding the correlation among HO-1 expression, cancer progression, patient prognosis, and outcome derive from immunohistochemistry studies on specimens from surgical patients. The data available in the literature are synthesized in Table 1 at the end of this paragraph. It is important to underline that, since Nrf2 is crucially involved in the regulation of HO-1 transcription, its expression has been considered as well. Both solid and hematopoietic malignancies have been taken into consideration, and the possible existence of negative association has also been analyzed.

### 5.1. HO-1 Expression and Disease Outcome

HO-1 expression in tumor mass is associated with poor prognosis/outcome and with high grade/stage in several types of tumors. In serous ovarian cancer, the association of HO-1 expression with FIGO stage III-IV and with poor overall survival has been proven [160]. In non-muscle-invasive bladder cancer (NMIBC), HO-1 expression is associated with grade 3, and poor prognosis or low recurrence/progression-free survival [161,162]. Similarly, in astrocytoma, high levels of HO-1 have been associated with tumor grade II and III and poor overall survival [163], and NSCLC at stage III-IV, high levels of HO-1 have been associated with high mortality risk and short overall survival [118]. In gallbladder cancer, the positivity for Nrf2, together with high expression of HO-1, has been shown to correlate with high grade/stage and poor prognosis [164], highlighting the role played by Nrf2 in the induction of HO-1 during tumor progression. Similar observations have been provided for clear cell renal cell carcinoma (ccRCC) [165], even though without correlation with tumor grade or stage. Indeed, patients with ccRCC showing high levels of HO-1 and Nrf2 have lower median survival time and shorter post-operative overall survival, with no proven correlation with tumor grade/stage.

In some studies, the expression level of HO-1 in tumors has been associated with clinical outcomes but without reference to the histopathological analysis. Thus, cholangiocarcinoma [166], acute myeloid leukemia (AML) [167], and neuroblastoma [168] show a correlation between high HO-1 expression and poor disease outcomes. Furthermore, HO-1 positivity in chronic myeloid leukemia [169], acute myeloid leukemia [170], and myelodysplastic syndrome [171] correlate with disease progression, resistance to therapy, and relapse.

### 5.2. HO-1 Expression and Tumor Grade/Stage

Vice versa, in other reports, HO-1 expression has been correlated with grade and stage and with invasion potential, but the clinical outcomes have not been analyzed. For instance, HO-1 overexpression in papillary thyroid cancer positively correlates with the TNM stage and cancer progression [172].

The intensity of HO-1 positivity has also been analyzed in order to find a possible correlation with the progression of a disease or with clinical outcomes. Interestingly, in NSCLC, the levels of HO-1 correlates with advanced stage (III-IV), T3, and T4 status and with lymph node metastasis; however, no association with overall survival has been demonstrated when patients were divided into two different subgroups related to HO-1 intensity of expression. Thus, no differences in patient survival were observed with regard to HO-1 intensity, highlighting that HO-1 positivity also at a low degree correlates with disease severity [98].

### 5.3. Correlation between HO-1 Expression and Tumor Markers

In many studies, HO-1 positivity has been correlated with other tumor markers. In localized prostatic cancer, HO-1 positivity associates with relapse frequency and PTEN deletion [173]. In NMIBC bladder cancers, HO-1 expression in tumor mass correlates with HIF-1α expression and microvessel density [123], and in particular, Nrf2 and HO-1 positivity correlates with HIF-1α, HIF-2α, and VEGF expression in the tumor, and with VEGF and interleukin levels in the plasma [124]. Similarly, in gastric cancer [174] and hepatocellular carcinoma [175], HO-1 positivity is associated with VEGF expression, poor differentiation, and microvascular density.

It is worth noting, in melanoma [176], thyroid cancer [172], and acute myeloid leukemia [167], HO-1 positivity correlates with the gain of function mutations of specific oncogenes B-Raf and RET. Moreover, in high-risk and very high-risk myelodysplastic syndrome, HO-1 expression correlates with overexpression of the enhancer of the zeste homologue 2 (EZH2) gene [171].

It is remarkable to note that HO-1 expression can be detected not only in tumor cells but also in cancer-associated cells, where it can contribute to the generation of a tumor-permissive environment. The number of HO-1 positive cancer-associated cells correlates with the tumor grade, metastatic competence, and neoangiogenesis. Indeed, in NMIBC bladder cancer HO-1 positivity has been detected not only in tumor cells but also in infiltrating fibroblasts and endothelial cells, in association with an increased risk of metastasis but without association to recurrence [177]. Further, high levels of HO-1 in infiltrating macrophages show a positive correlation with vascular density and high tumor grade in glioblastoma [178], with stage II, lymph node metastasis, and poor prognosis in colorectal cancer [179], and with a high Gleason score and bone metastasis in prostate cancer [117]. High HO-1 expression in lymphocyte T_reg_ shows a correlation with a high tumor grade in glioma [137].

### 5.4. Contrasting Evidence

Although a great deal of literature highlights the correlation between HO-1 overexpression and cancer progression and often with the poor clinical outcomes, it seems important to consider that opposite evidence has also been provided. Indeed, it has been demonstrated that high HO-1 expression level correlates with a better prognosis and better overall survival in colorectal cancer [180,181], in gastric cancer [182], in small intestinal adenocarcinoma [183], and in oral squamous carcinoma [184].

An important observation concerning HO-1 subcellular localization comes from three different studies on head and neck cancer [185], breast cancer [186], and colorectal cancer [187] that analyzed the correlation of histological features with HO-1 positivity in cytosol or nuclei. In these studies, high expression of HO-1 in cytosol correlated with low grade and differentiation without correlation with invasiveness. However, nuclear localization of HO-1 was associated with a high grade and poor differentiation. Moreover, in breast cancer, Gandini showed that cytosolic HO-1 is enzymatically active, while the nuclear form is truncated and with no catalytic activity [186]. These observations appear to be interesting and helpful in understanding the contrasting observation of the role of HO-1 in tumor progression and lead to speculation that HO-1 pro- or antitumor activity may depend on its subcellular localization and catalytic activity.

**Table 1:** Correlation among HO-1 expression, aggressiveness, and outcomes in histological specimens.

Tumor	HO-1	Nrf2	Grade and Stage	Additional Markers	Metastasis, Lymph Node, Angiogenesis	Clinical and Pathological Features	Disease Outcome/Prognosis	Ref.
**Positive correlation among HO-1 expression and tumor aggressiveness/poor prognosis**								
**-Solid tumors**								
Astrocytoma	High level	n.e.	Grade II and III	n.e.	n.e.	n.e.	Poor OS	[163]
Clear cell renal cell carcinoma	High level	High level	No correlation with ISUP grade and T stage	n.e.	No correlation with lymph node metastasis	No significant correlation with age, gender	Poor prognosis Low MST Low post operative OS	[165]
Colangiocarcinoma	High level	n.e.	n.e.	n.e.	No association with metastasis	No significant association with age, gender, histological type	Poor OS	[166]
Gastric cancer	High level	High level	Poor differentiated tumors	Positive correlation with VEGF	Positive correlation with MVD	n.e.	n.e.	[174]
Gallbladder cancer	High level	High level	Moderately differentiated and poorly differentiated tumors (G2-G3) Correlation with Nevin classification (III-IV-V)	Positive correlation with MRP3	Metastasis	No significant correlation with gender, age, and histology type (SCC and AD)	Poor OS	[164]
Hepatocellular carcinoma	High level	n.e.	Poor differentiated tumors Edmondson-Steiner grade 2–4	n.e.	Microvascular and capsular invasion	High levels of preoperative AFP	No significant correlation with OS and recurrence	[175]
Hormone refractory prostate cancer	High level	n.e.	n.e.	n.e.	n.e.	Cancer progression	n.e.	[188]
Laryngeal cancer	High level	High level	No correlation with tumor stage (clinical stage III and IV), size tumor	High level Keap1 and NQO1	No correlation with lymph node metastasis	No correlation with age	n.e.	[189]
Melanoma	High level	n.e.	n.e.	Positive correlation with B-Raf and ERK	n.e.	n.e.	n.e.	[176]
Neuroblastoma	High level	n.e.	n.e.	n.e.	n.e.	n.e.	Poor OS	[168]
Non-muscle-invasive bladder cancer	High level	n.e.	Tumor grade G3 tumor stage pT1	Ki-67 and p53	n.e.	No significant correlation with age and gender	Poor prognosis No correlation with RFS and PFS	[161]
	High level	n.e.	Tumor grade G3 Tumor stage T1	Positive correlation with S100A4	Lymph vascular invasion	n.e.	Low RFS Low PFS	[162]
	High level	n.e.	n.e.	Positive correlation with HIF-1α	High MVD	n.e.	n.e.	[123]
	High level	High level	n.e.	Correlation with HIF-1α, HIF-2α, VEGF	n.e.	Increased serum/plasma level of IL-6, IL-8, VEGF	n.e.	[124]
Non-small cell lung cancer	High level	n.e.	Stage III-IV	Positive correlation with MMP-9	High metastatic rate	No correlation with age and gender	Poor prognosis Low OS High mortality risk	[118]
	High level	n.e.	Stage III-IV T status (T3-T4)	n.e.	Lymph node metastasis	No correlation with gender	No significant difference in patient survival between high and low staining group	[98]
Ovarian cancer	High level	n.e.	Serous undifferentiated tumors Correlation with FIGO stage (III-IV)	n.e.	Lymph node metastasis	Non optimal-debulking	Poor OS	[160]
Prostate cancer	High level	n.e.	Localized tumor	PTEN deletion	n.e.	n.e.	Relapse after radical prostatectomy	[173]
Thyroid cancer	High level	n.e.	Positive correlation with TNM (1,2,3,4) and with MACIS score	BRAFV600E mutation	No significant association with lymph node metastasis	Correlation with age and tumor aggressiveness	n.e.	[172]
**-Hematopoietic tumors**								
Acute myeloid leukemia	High level	n.e.	n.e.	Positive correlation with HIF-1α and GLUT-1	n.e.	n.e.	Correlation with relapse and refractory	[170]
	High level	n.e.	Correleation with M5 patients	Correlation with RET gene	n.e.	Correlation with leukocytosis at diagnosis	n.e.	[167]
Chronic myeloid leukemia	Higher level in peripheral blood cells	n.e.	n.e.	n.e.	n.e.	Tumor progression	Correlation with relapse	[169]
Myelodysplastic Syndrome	High level	n.e.	Correlation with high-risk and very high-risk patients	Positive correlation with EZH2	n.e.	Progression to AML and decreased response to decitabine	n.e.	[171]
**Positive correlation among HO-1 expression in tumor-associated cells and tumor aggressiveness/poor prognosis**								
Colorectal cancer	High level in cancer cells and in macrophages	n.e.	Stage III	n.e.	Lymph node metastasis	No significant difference between the HO-1-positive and negative with gender, age, tumor size, histological type, and depth of tumor invasion	Poor prognosis Short DSF	[179]
Glioblastoma	High level in infiltrating macrophages	n.e.	Grade IV	n.e.	Positive correlation with vascular density	n.e.	n.e.	[178]
Glioma	HO-1 positive Treg	n.e.	Correlation with grade glioma (II-III-IV)	n.e.	n.e.	n.e.	n.e.	[137]
Non-muscle-invasive bladder cancer	High level in cancer cells and fibroblast-like, tumor- infiltrating, and endothelial cells	n.e.	Correlation with high grade tumors and with stage (T1)	COX-1	MVD, LVD, PI, increased risk of metastasis	No association with age and gender	No association with recurrence	[177]
Prostate cancer	HO-1 positive macrophages infiltrate and in bone metastasis	n.e.	High-grade tumors Gleason score 7–10	n.e.	Bone metastasis	n.e.	n.e.	[117]
**Negative correlation among HO-1 expression and tumor aggressiveness/poor prognosis**								
Colorectal cancer	High level	n.e.	Invasive CRC	Significant correlation with K-ras	n.e.	Significant correlation with normal CEA level	Better prognosis, increased MTS	[181]
	High level	n.e.	n.e.	n.e.	Low vascular invasion and lymph node metastasis	n.e.	Better survival rate	[180]
Gastric cancer	High level	n.e.	Well and moderate differentiated	n.e.	Negative lymph node metastasis	n.e.	Better prognosis	[182]
Oral squamous cell carcinoma	High level	n.e.	Well-differentiated Grade G1 No association with T stage	n.e.	Low lymph node metastasis	No association with age and sex No association with clinical stage	n.e.	[184]
Small intestinal adenocarcinoma	High level	n.e.	Low T stage (T1, T2, T3)	n.e.	Low pancreatic invasion	n.e.	Tend to have longer OS (difference not significative)	[183]
**Different correlation among HO-1 expression and tumor aggressiveness/poor prognosis depending on HO-1 subcellular localization**								
Breast cancer	High level in malignant epithelial cells	n.e.	Grade I-II (>80%)	Positive correlation with E-cadherin	Negative correlation with lymph node metastasis	Reduced tumor size	Longer OS with increased MST	[186]
Colorectal cancer	High level in cancer cells and in stromal cells (fibroblasts, neutrophils, and macrophages)	n.e.	Well-differentiated adenocarcinoma Nuclear HO-1 localization in moderate and poor differentiated No association with TNM	n.e.	No correlation with lymph node and liver metastasis	n.e.	n.e.	[187]
Head and neck squamous cell carcinoma	High level	n.e.	High rate of HO-1 positivity in well-differentiated and moderately differentiated (<90%) Poor-differentiated high rate of nuclear HO-1	n.e.	n.e.	No association with age, gender, tumor location	n.e.	[185]

Tumors are listed alphabetically. List of table abbreviations. n.e., not evaluated; AD, adenocarcinoma; AFP, alpha feto protein; CEA, carcinoempryonic antigen; ISUP, International Society of Urologic Pathologists; LVD, lymph vascular density; MTS, median survival time; MVD, microvascular density; OS, overall survival; PI, proliferation index: PFS, progression free survival; RFS, recurrence free survival; SCC, squamous cell carcinoma.

## 6. HO-1 and Tumor Therapies

It has been widely reported that the induction of HO-1 in response to anticancer treatments can attenuate the efficacy of therapy, increasing cancer cell survival. Indeed, HO-1 expression is increased in response to different chemotherapeutic agents that act through the imbalance of intracellular oxidative state. For instance, in neuroblastoma cells, HO-1 expression is induced by exposure to etoposide through the activation of Nrf2 [145], and by the exposure to proteasome inhibitors bortezomib or carfilzomib [148,149,150], and mediates cell survival. To note, doxorubicin or pharmorubicin promote HO-1 expression increasing cell survival in breast cancers through the activation of Src/STAT3 or PI3K/AKT, respectively [146,147].

Remarkably, HO-1 induction mediates cancer cell resistance not only to chemotherapeutic agents but also to radio-, photodynamic-, and non-thermal-plasma (NTP) therapies, as demonstrated in non-small cell lung carcinoma [152,153,154].

As far as hematological malignancies are concerned, HO-1 expression significantly increases in myeloid neoplasms both in chronic and acute myeloid leukemia. Its overexpression occurs mainly after therapeutic intervention and induces chemoresistance. Recently, it has been demonstrated that PI3K/AKT-dependent HO-1 induction drives drug resistance to imatinib in CML [190] as well as to panobinostat in AML [191] by modulating the expression of HDACs. HO-1 overexpression enhances the viability and decreases the apoptotic rate in AML cell lines treated with cytarabine. Accordingly, the derived xenograft mouse model shows a significantly shorter survival and a great extent of organ invasion, while HO-1 down regulation significantly increases the survival rate [192]. Moreover, HO-1 up-regulation in myelodysplastic syndromes is closely related to resistance to decitabine-induced apoptosis [193], and in multiple myeloma, HO-1 up-regulation is involved in bortezomib chemoresistance [194].

In this context, pharmacological and genetic tools to reduce HO-1 activity have been proposed, and their use has been hypothesized in therapy, as described later and summarized in Table 2.

### 6.1. Inhibition of HO-1 by Pharmacological Compounds

Among the pharmacological tools, metalloporphyrins and imidazole-based compounds are the most well-known and have been recently reviewed [195].

Briefly, metalloporphyrins represent the first generation of HO-1 inhibitors and include deuteroporphyrin, mesoporphyrin, and protoporphyrin [196]. Structurally similar to heme, metalloporphyrins strongly inhibit HO-1 by a competitive mechanism [197]. The most used metalloporphyrins are ZnPPIX, SnPPIX, and SnMPIX, and their efficacy in favoring conventional tumor therapies has been widely demonstrated in vitro and in vivo. For instance, ZnPPIX favors the sensitivity of nasopharyngeal carcinoma cells to radiotherapy [198] and of neuroblastoma to glutathione depletion and etoposide [145]. Moreover, ZnPPIX sensitizes C-26 colon and MDAH2774 ovarian carcinoma cells to photodynamic therapy-mediated cytotoxicity [199] and increases the effects of cisplatin in liver cancers [143]. It has also been demonstrated that treatment with ZnPPIX reduces cell growth in hepatoma, sarcoma, lung cancer, and B cell lymphoma [52,125]. Furthermore, in melanoma cells, SnPPIX enhances the efficacy of photodynamic therapy [200] and in BRAF^V600^-mutated melanoma cells SnMPIX increases cell death induced by vemurafenib/PLX4032 [140].

Unfortunately, metalloporphyrins are able to act on other heme-dependent enzymes, such as nitric oxide synthase (NOS), sGC, and cytochrome P450 [201,202]. Moreover, even though they efficiently inhibit HO-1 activity, they can often favor HO-1 protein synthesis, as demonstrated in liver cells and fibroblasts, and more recently, in prostate cancer PC-3 cells by a compensatory mechanism [203,204,205]. Of note, another important disadvantage of using metalloporphyrins is related to their photo reactivity, which is responsible for side effects and even tissue and organ damage [196]. Another strong drawback for the potential clinical use of some metalloporphyrins (e.g., ZnPPIX) is represented by their poor solubility in aqueous solutions, which limits translational applicability. However, this inconvenience has been overcome by conjugation with specific molecules, e.g., polyethylene-glycol or amphiphilic styrene-maleic acid copolymer, generating water-soluble molecules [206,207,208,209,210].

Imidazole-based compounds represent the second generation of HO-1 inhibitors. These molecules are non porphyrin-based and non competitive water-soluble inhibitors of HO-1 and exhibit low or even no inhibitory action on NOS, sGC, and CYP [211,212]. The first reported was Azalanstat [213], but other molecules and novel azole-based compounds derived from the structural modification of Azalanstat have been recently discovered [214,215]. Imidazole-based compounds have shown potent antitumor activity in prostate and breast cancer cell lines [216]; in a preclinical model of hormone-refractory prostate cancer, the small molecule imidazole-derived OB-24 acts in synergism with the conventional chemotherapy drug Taxol, preventing tumor growth and formation of lymph node and lung metastasis [188]. However, imidazole-based compounds have not been tested in clinical studies so far.

### 6.2. Inhibition of HO-1 by RNA Interference and CRIPR/Cas9 Technology

With regard to genetic tools to modulate HO-1 activity, the most consistent data derive from studies on RNA interference, including small interfering RNA and short hairpin RNA, able to inhibit HO-1 activity by targeting HO-1 transcription and consequently protein synthesis. Thus, HO-1 silencing increases the effect of chemotherapeutic drugs in pancreatic cancer [217], neuroblastoma [148,149], and melanoma cancer cells [140], as well as in myeloid leukemia [170]. Moreover, HO-1 silencing sensitizes cancer cells to apoptosis, as demonstrated in lung, colon, and leukemic cancer cells [195]. Similar results have been obtained in an in vivo experimental mouse model of hepatocellular carcinoma, where injection of siRNA-HO-1 results in the diminished growth of the tumor [218]. Furthermore, HO-1 is considered a survival factor in ALL, regardless of Philadelphia chromosome positivity; indeed, the down-regulation of HO-1 expression by siRNA increases apoptosis and arrests cell growth [219]. Consistently, in chronic lymphocytic leukemia (CLL), it has been demonstrated that HO-1 silencing directly leads to apoptosis of MEC-1 cells and enhances the effects of the combined therapy fludarabine plus entinostat [220].

A new approach in the inhibition of HO-1 activity is represented by genetic ablation of HO-1 with the CRISPR/Cas9 editing system. It has been recently demonstrated that homozygous HO-1 knock-out in BRAF-WT melanoma cells is able to decrease clone formation and to lower tumor cell growth [176]; further, in pancreatic ductal adenocarcinoma cells, HO-1 CRISPR/Cas9 is able to suppress cell proliferation and improve the efficacy of gemcitabine treatment [151]. Importantly, in in vivo experiments on C57/BL6 mice, HO-1 CRISPR/Cas9 editing blocks lymphocyte B development [221].

**Table 2:** HO-1 inhibitory tools.

Pharmacological Inhibitors		Benefits	Drawbacks	Ref.
	**Porphyrin-Based Compounds**			
	Metalloporphyrins - Zinc II protoporphyrin IX (ZnPPIX) - Tin protoporphyrin IX (SnPPIX) - Tin mesoporphyrin IX (SnMPIX)	- Competitive inhibitors - Well proved activity in vitro and in vivo	- Non selective on HO-1 isoform - Active on other heme-dependent - enzymes (NOS, sGC, and CYP) - HO-1 inducers - Photo reactive - Poor soluble	[196,201,202]
	**Modified protoporphyrins** - Polyethylene-glycol (PEG-ZnPPIX) - Amphiphilic styrene-maleic acid copolymer (SMA-ZnPPIX)	- Water-soluble		[206,207,208,209,210]
	**Imidazole-based compounds**			
	- Azalanstat - Other imidazole-derived compounds - (OB-24)	- Non competitive inhibitors - Selective on HO-1 isoforms - Limited inhibitory activity on NOS, sGC, and CYP - Water-soluble	- Not well studied and not tested in clinical trials	[211,212]
**Genetic tools**				
	Small interfering RNA and short hairpin RNA	- Specific targeting HO-1 mRNA	- Limited therapeutic application (delivery methods)	[195]
	CRISPR/Cas9	- Genetic ablation of HO-1 gene - Stable knock-down - High efficiency of HO-1 inhibition	- Limited therapeutic application (delivery methods)	[195]

### 6.3. Induction of HO-1 as a Therapeutic Strategy

Thus, a great deal of literature shows a direct correlation between the overexpression of HO-1 and the gain of resistance of cancer cells and tumor progression. However, it must be taken into account that in some tumors, the over expression of HO-1 exerts opposite effects by inhibiting tumor growth and cancer progression. In particular, it has been shown in some types of prostate cancer that HO-1 expression and carbon monoxide generation are associated with significant inhibition of cell proliferation and invasiveness [96]. Moreover, in non-small-cell lung carcinoma NCI-H292 cells, the stable HO-1 overexpression is able to up-regulate tumor-suppressive miRNAs and to down-regulate the expression of oncomirs and angiomirs, leading to the inhibition of cell proliferation, invasiveness, and angiogenesis [222]. It has been highlighted that this tumor-suppressive phenotype is characterized by the attenuation of the metastatic potential mainly by down regulating MMP-9 and MMP-13 [223]. Similarly, stable overexpression of HO-1 retards hepatocellular carcinoma progression [224]. The antitumorigenic effects of HO-1 have also been demonstrated in human and rat breast cancer, where its overexpression correlates with inhibition of cell proliferation [225] and in pancreatic and prostate cancer, where it is associated with a decrease in cell proliferation and invasiveness by a down regulation of the proangiogenic mediators VEGF and MMP-9 [97,195,226]. In this context, the induction of HO-1 has been proposed to increase conventional cancer therapies, and some “natural” compounds derived from plants have shown interesting properties. In colorectal cancer, it has been demonstrated that treatment with extracts from *Sageretia thea*, a medicinal plant used for treating hepatitis and fevers in Korea and China, decreases cell viability by inducing GSK3β-dependent cyclin D1 degradation and increasing HO-1 expression via activation of Nrf2 [227]. In addition, Ginnalin A, a polyphenolic compound isolated from red maple (*Acer rubrum*), inhibits cell viability and colony formation in colorectal cancer, inducing cell cycle arrest by activating the Nrf2/HO-1 pathway through the up-regulation of p62 and the inhibition of Keap1 [228]. Similarly, treatment with fisetin, a bioactive flavonol molecule abundantly found in strawberries, decreases the level of MMPs and cell migration in metastatic breast cancer with a mechanism depending on Nrf2 nuclear translocation and HO-1 up-regulation [229].

Since ferroptosis may be a way to kill cancer cells, and it can be enhanced by HO-1 overactivation, the pharmacological induction of HO-1 has been proposed. Indeed, HO-1-dependent intracellular Fe^2+^ overload induces lipid peroxidation and triggers a noncanonical ferroptosis [230]. Phytochemicals are often used for this purpose [231]. Neuroblastoma cell treatment with withaferin A, a steroidal lactone derived from *Withania somnifera* (Indian ginseng), directly targets Keap1, leading to Nrf2 release and HO-1 up-regulation and consequently increasing intracellular Fe^2+^ and inducing ferroptosis [232]. Similarly, in human colon cancer cells, a high concentration of extract of *Betula etnensis* extract induces HO-1 leading to ferroptotic cell death through an increase of ROS production and in lipid peroxidation mediated by iron accumulation [233]. Moreover, HO-1 up-regulation has been proved to be the primary factor for curcumin-induced ferroptosis in human breast adenocarcinoma-derived MCF7 cells and in human triple-negative MDA-MB-231 cell line [234]. In addition, β-elemene, a sesquiterpene found in a variety of plants, is able to induce ferroptosis by enhancing HO-1 activity in KRAS mutant colorectal HCT116 cancer cells [235]. In addition, in this work, the presence of possible side effects of β-elemene were tested in the derived orthotopic murine colon cancer model, and no toxicity was found relatively the different organs analyzed (lung, heart, liver, kidney, and spleen) by H&E staining.

Thus, the evaluation of HO-1 expression in cancer samples from patients may help to define a therapeutic strategy where inhibition or induction of HO-1 could improve the efficacy of the standard antineoplastic therapy used.

## 7. Future Perspectives and Conclusions

The chance to analyze HO-1 expression in cancer patients seems to be a useful tool to improve tumor diagnosis and to better define prognosis and therapy. On the one hand, the analysis of (GT)n length polymorphisms seems a very promising approach to assess the risk of treatment failure as recently proved in ALL patients carrier of short (GT) repeats [60]. On the other hand, the characterization of HO-1 expression in tumors may be a useful tool to improve tumor diagnosis and prognosis because it can correlate with tumor grade/stage, invasiveness, and clinical outcomes. However, contrasting data are reported, and larger analyses need to be performed. Importantly, it has been recently highlighted the role played by the truncated form of HO-1 in favoring cell growth, opening to a new scenario in which HO-1 can be involved in tumor biology [92].

As a future perspective, in order to better assess tumor progression, the correlation between tissue expression of HO-1 and its levels in a blood sample could be taken into consideration, even though no evidence has been reported so far. However, the analysis of HO-1 level may be proposed in other biological fluids such as urine, peritoneal or pleural fluids, if directly related to the tissue bearing neoplastic cells. It is important to remember that in other diseases, HO-1 levels in bio fluids correlate with HO-1 expression levels in tissues [103].

**Figure 2:**
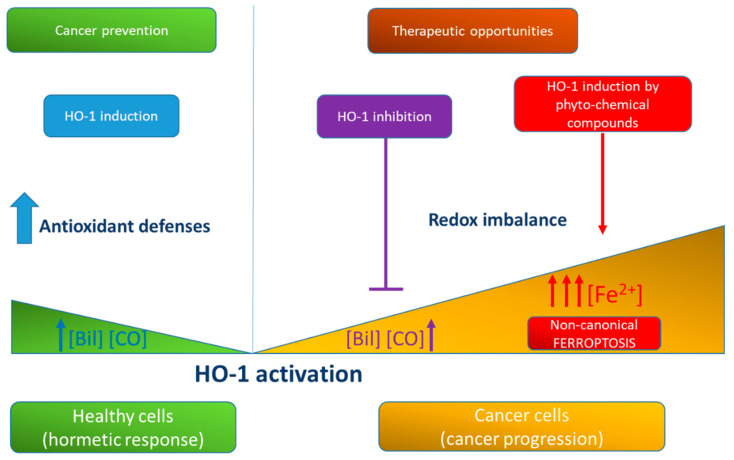
Schematic representation of the effects of HO-1 activation and generation of its metabolic products in healthy and cancer cells. HO-1 activation is involved in antioxidant defenses and in healthy cells promotes the hormetic response and cancer prevention through the generation of bilirubin and CO. In cancer cells, HO-1 favors cancer progression, and its inhibition represents a therapeutic opportunity. However, also HO-1 over-activation can be proposed as a therapeutic option, as it can favor unconventional ferroptosis through the accumulation of pro-oxidant-free iron.

Moreover, a great amount of data support the efficacy of HO-1 modulation in order to improve cancer response to therapies (Figure 2). Different approaches have been proposed, using either pharmacological agents or genetic tools. Unfortunately, concerning HO-1 pharmacological inhibitors, the translational applicability is not completely elucidated, even though both SnPPIX and SnMPIX have been already tested in humans [236] and approved for the treatment of hyperbilirubinemia [237]. Instead, genetic tools have been tested only in experimental animal models. Therefore, HO-1 modulation may represent an important strategy also to prevent cancer immune escape. However, we must consider that, so far, little data in the literature are available on the role played by HO-1 in the function of tumor-related immune cells. This is still an open field of research.

Conversely, molecules able to induce HO-1 may be used in order to favor cancer cell death due to iron imbalance. About this issue, as mentioned before, many natural compounds have been tested and showed their efficacy in this sense, but even in this case, translational applicability in humans seems to be still far away.

In conclusion, a deeper investigation of the specific multifaceted role played by HO-1 in different types of cancers, in the tumor microenvironment and bio fluids is needed in order to customize therapy and improve the outcome of cancer patients. Thus, HO-1 could become in the future an important clinical tool for cancer management.

## Abbreviations

ALL	acute lymphoblastic leukemia
AML	acute myeloid leukemia
BR	bilirubin
BVRA	biliverdin reductase A
ccRCC	clear cell Renal cell carcinoma
CML	chronic myeloid leukemia
DC	dendritic cells
EV	extracellular vescicles
HO-1	heme oxygenase 1
MAPK	mitogen-activated protein kinase pathway
NK	natural killer cells
NMIBC	non-muscle-invasive bladder cancer
NOS	nitric oxide synthase
PMBC	peripheral blood mononuclear cells
ROS	reactive oxygen species
sGC	soluble guanylyl cyclase
SnMPPIX	tin mesoporphyrin IX
SnPPIX	tin protoporphyrin IX
SSP	signal peptide peptidase
VSMC	vascular smooth muscle cells
ZnPPIX	zinc(II) protoporphyrin IX
